# Case Report: A clinical case of postoperative podaponeurotic abscess caused by *Actinomyces odontolyticus*

**DOI:** 10.3389/fmed.2026.1792898

**Published:** 2026-04-30

**Authors:** Alyona Lavrinenko, Nurkassi Abatov, Ruslan Badyrov, Nurlan Urazbayev, Aidana Turemuratova, Dias Yesniyazov, Marina Izmailovich

**Affiliations:** 1 Institute of Life Sciences, Karaganda Medical University, Karaganda, Kazakhstan; 2 Department of Surgery, Karaganda Medical University, Karaganda, Kazakhstan; 3 Clinic of the Medical University, Karaganda Medical University, Karaganda, Kazakhstan; 4 Department of Internal Medicine, Karaganda Medical University, Karaganda, Kazakhstan

**Keywords:** abscess, *Actinomyces odontolyticus*, appendicitis, postoperative abscess, soft tissue infection

## Abstract

*Actinomyces odontolyticus* is an uncommon cause of soft tissue infection and is usually regarded as a commensal microorganism of the oropharyngeal and gastrointestinal microbiota. This article reports the first documented case in the Republic of Kazakhstan of a postoperative wound infection caused by *A. odontolyticus* following surgery for acute phlegmonous appendicitis. This case emphasizes the diagnostic importance of including Actinomyces species in the differential diagnosis of postoperative soft tissue infections, particularly in cases of atypical wound healing.

## 1 Introduction

*Actinomyces odontolyticus* is a Gram-positive, facultative anaerobic bacterium belonging to the genus Actinomyces. It is a normal inhabitant of the mucosal surfaces of the oral cavity, upper respiratory tract, and gastrointestinal tract. However, when the integrity of mucosal barriers is compromised, this microorganism can become pathogenic, causing various infections, including abscesses, bacteremia, and soft tissue infections (1–3).

In postoperative patients, persistent low-grade fever, poor response to empirical antibacterial therapy, and signs of deep wound infection may indicate an atypical pathogen requiring microbiological verification and source control. In this context, delayed recognition of uncommon anaerobic microorganisms may complicate postoperative recovery. An expanded anaerobic workup and advanced identification methods should be considered in postoperative patients with persistent fever, inadequate response to empirical antibacterial therapy, and imaging findings suggestive of deep-space or postoperative wound infection. Similarly, previous case reports of uncommon postoperative or procedure-related infections have shown that failure of empirical therapy, followed by imaging, drainage, and microbiological confirmation, may be critical for establishing the diagnosis and guiding effective treatment (4, 5).

Postoperative infections caused by *Actinomyces odontolyticus* have been only rarely reported in the literature, with most published cases involving cervicofacial or pulmonary sites rather than postoperative soft tissue infections. The diagnosis of Actinomyces-related infections is often complicated by nonspecific clinical manifestations, poor response to empirical therapy, and slow bacterial growth on conventional culture media. Therefore, matrix-assisted laser desorption/ionization time-of-flight (MALDI-TOF) mass spectrometry and molecular genetic methods play an important role in accurate pathogen identification (6, 7).

To our knowledge, this is the first documented case in Kazakhstan of postoperative soft tissue infection caused by *Actinomyces odontolyticus*.

## 2 Case presentation

A 55-year-old female was admitted to the Emergency Department of Karaganda Medical University Hospital (Karaganda, Kazakhstan) with complaints of acute right lower quadrant abdominal pain, dry mouth, and low-grade fever (up to 37.5 °C). The pain had started three days before admission, initially diffuse, and later localized to the right iliac region.

The patient had no history of chronic diseases, prior surgical interventions, or immunosuppressive conditions. Screening for infectious diseases, including hepatitis B, hepatitis C, and HIV, was negative. The allergic history was unremarkable. The patient had a body mass index of 28.2 kg/m^2^, corresponding to overweight, which is a potential risk factor for postoperative complications. No history of smoking, regular medication use, or other relevant social risk factors was reported.

On admission, the patient was in a stable general condition with moderate pain. There were no signs of hemodynamic instability or systemic intoxication. Vital signs were as follows: blood pressure 120/80 mmHg, heart rate 78 beats per minute, respiratory rate 18 breaths per minute, and body temperature 37.5 °C.

On physical examination, localized tenderness on palpation was detected in the right iliac region, with positive Rovsing’s, Sitkovsky’s, and Bartomé–Michelson signs. Signs of peritoneal irritation were absent. Laboratory tests revealed an elevated C-reactive protein level (6 mg/L; reference < 5 mg/L), while the white blood cell count remained within normal limits.

**Figure 1:**
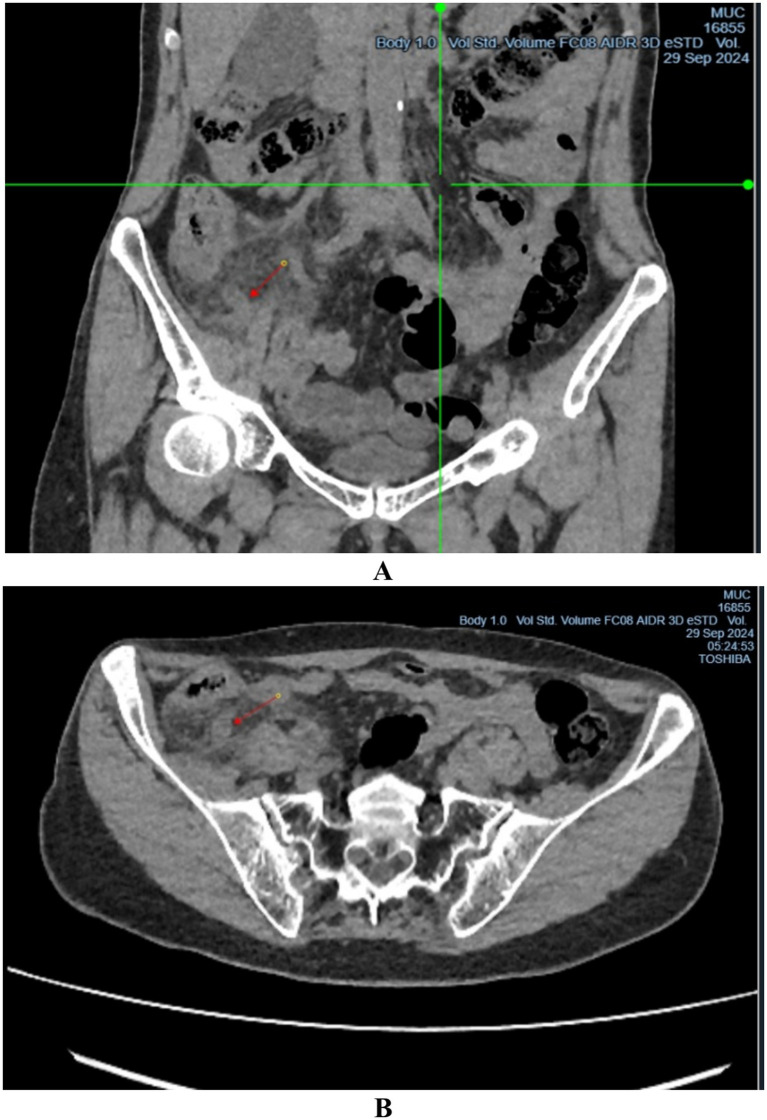
CT image of acute appendicitis, presented in the: **(A)** frontal projection; **(B)** axial projection.

Computed tomography (CT) of the abdomen demonstrated findings consistent with acute appendicitis (Figures 1A,B). The patient underwent open appendectomy via an oblique Volkovich–Dyakonov incision, and acute phlegmonous appendicitis was confirmed intraoperatively. In our department, laparoscopic appendectomy is generally the preferred approach for acute appendicitis because it is associated with less postoperative pain, shorter hospital stay, and fewer wound-related complications. In the present case, however, an open appendectomy was performed because of technical problems with the laparoscopic equipment at the time of surgery. No specific wound protection device was used intraoperatively; however, standard aseptic measures, including careful skin preparation, sterile draping, and routine postoperative wound care, were strictly followed.

In the immediate postoperative period, the patient remained hemodynamically stable, with blood pressure 130/80 mmHg, heart rate 84 beats per minute, respiratory rate 17 breaths per minute, and body temperature 36.8 °C. The general condition was of moderate severity, consistent with the early postoperative state.

**Figure 2:**
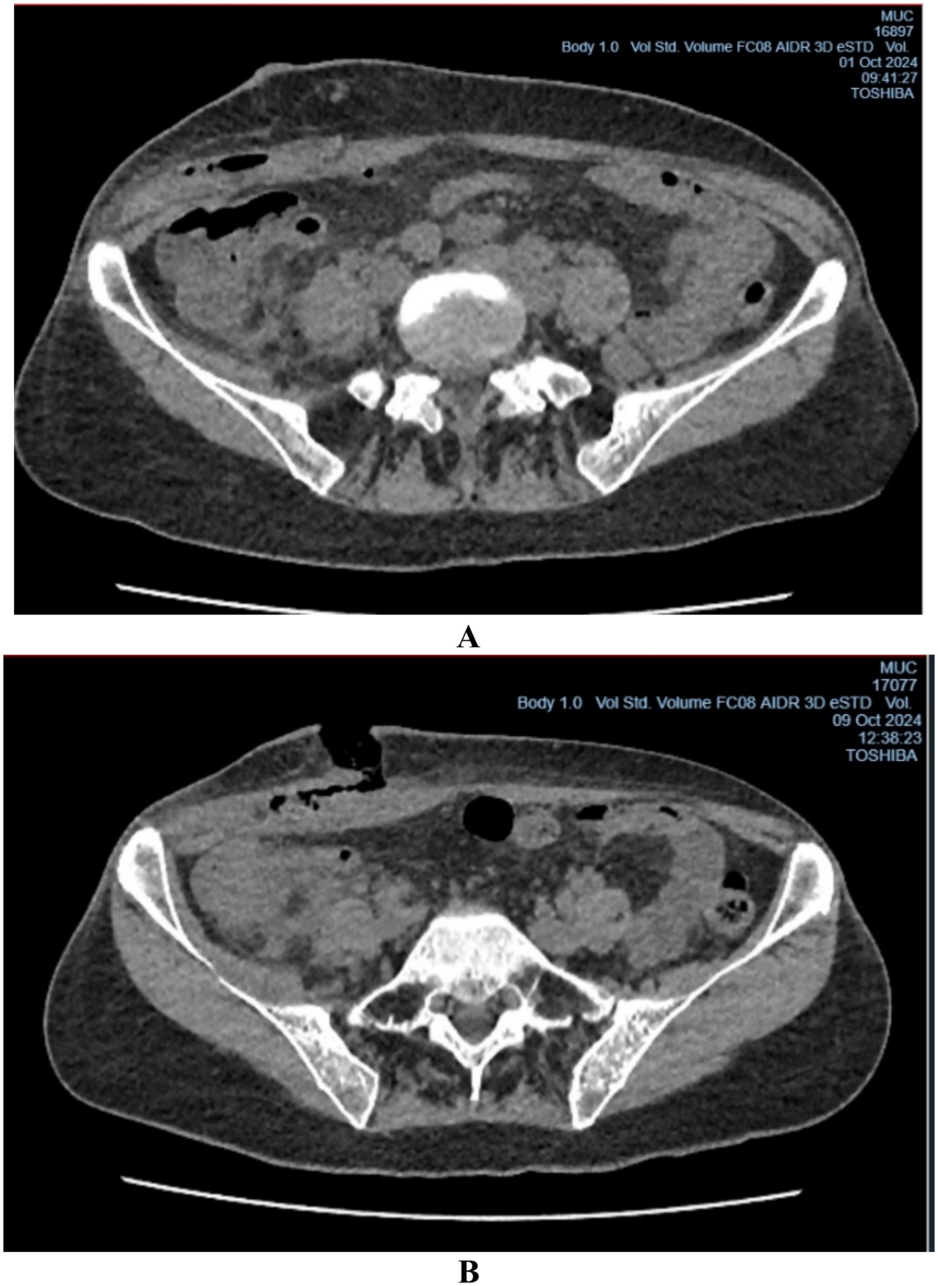
CT image: **(A)** a subaponeurotic abscess in the area of the postoperative wound in the right iliac region; **(B)** after the drainage of the subaponeurotic abscess.

In the early postoperative period, the patient developed persistent low-grade fever (37.5–38.0 °C) and localized tenderness at the surgical site without marked superficial inflammatory changes such as hyperemia or wound dehiscence. At that time, vital signs were as follows: blood pressure 110/70 mmHg, heart rate 88 beats per minute, respiratory rate 20 breaths per minute, and body temperature up to 38.0 °C. The patient remained hemodynamically stable; however, the general condition was of moderate severity due to pain and signs of systemic inflammatory response. Laboratory evaluation revealed leukocytosis (11.2 × 10^9^/L; reference range 4–9 × 10^9^/L) with neutrophilia (neutrophils 78%; reference 35–70%; band neutrophils 4%; reference 1–5%). The lack of response to empirical antibacterial therapy (third-generation cephalosporin, 1.0 g intramuscularly twice daily) raised suspicion of a deep postoperative infection, specifically a subaponeurotic abscess, despite the absence of pronounced superficial wound changes (Figures 2A,B).

**Figure 3:**
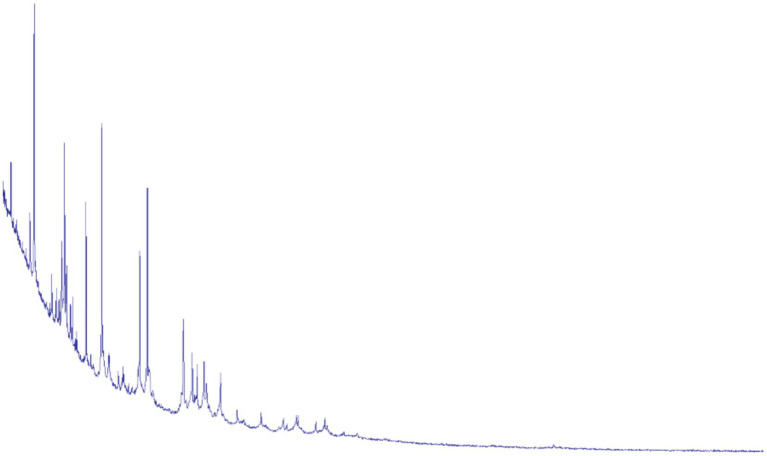
*Actinomyces odontolyticus* peaks on MALDI-TOF.

On postoperative day six, the patient underwent surgical wound revision under local anesthesia using 0.5% novocaine (30 mL). A purulent cavity beneath the aponeurosis of the external oblique abdominal muscle was identified and drained (Figure 3). At the time of revision, purulent discharge from a deep subaponeurotic cavity was identified, without clear signs of superficial wound infection, consistent with a localized deep soft tissue abscess. A specimen was submitted for microbiological analysis to the research laboratory of Karaganda Medical University.

Purulent material was collected for microbiological examination at the time of surgical wound revision on postoperative day six. In accordance with standard surgical practice, empirical antibacterial therapy was initiated prior to the availability of culture results, given the clinical signs of postoperative infection.

Following definitive pathogen identification and antimicrobial susceptibility testing, the treatment strategy was adjusted accordingly, which was associated with rapid clinical improvement, including resolution of fever and regression of local inflammatory signs. This reflects routine clinical practice, where empirical therapy is initiated prior to microbiological confirmation.

The total duration of hospitalization was 17 days. Clinical improvement was observed within several days after initiation of targeted antimicrobial therapy, with subsequent normalization of laboratory parameters. The wound healed by secondary intention with active granulation tissue formation. No recurrence or need for re-intervention was observed during follow-up.

Timeline of clinical events:

- Day −3: Onset of abdominal pain.
- Day 0: Hospital admission and appendectomy.
- Postoperative days 1–5: Persistent fever and local pain.
- Postoperative day 6: Surgical revision and drainage.
- Post-drainage: Identification of *Actinomyces odontolyticus* and targeted therapy.
- Follow-up: Clinical recovery and wound healing.

### 2.1 Microbiological findings

The specimen was cultured on blood agar using standard microbiological techniques. After incubation, colonies morphologically consistent with Actinomyces species were detected, with a colony-forming unit count of 10^5^ CFU/mL. MALDI-TOF mass spectrometry enabled definitive identification of *Actinomyces odontolyticus* (Figure 3).

Antimicrobial susceptibility testing demonstrated sensitivity to penicillin, amoxicillin, cefoxitin, ceftriaxone, imipenem, fusidic acid, tetracycline, levofloxacin, and linezolid, while resistance to clindamycin and azithromycin was observed.

Based on susceptibility results, the patient was treated with ceftriaxone 1.0 g intramuscularly twice daily, combined with daily antiseptic wound dressings. Marked clinical improvement was observed, including resolution of fever and regression of local inflammatory signs. Follow-up laboratory tests demonstrated normalization of leukocyte counts (7 × 10^9^/L). The wound healed by secondary intention with active granulation tissue formation. Repeated bacteriological cultures were negative for *A. odontolyticus*, confirming successful eradication of the pathogen.

## 3 Discussion

Abdominal actinomycosis is a rare infection caused by facultative anaerobic, Gram-positive bacilli of the genus Actinomyces, which normally colonize the oral cavity, gastrointestinal tract, skin, and genitourinary system (8). In the present case, disruption of mucosal integrity was more likely related to the underlying acute phlegmonous appendicitis than to the surgical procedure itself. Inflammatory destruction of the appendiceal wall may have facilitated bacterial translocation and subsequent infection, whereas surgery may have acted only as an additional contributing factor rather than the primary cause. Abdominal actinomycosis is most commonly associated with appendicitis or prior abdominal surgery (9, 10). A major diagnostic challenge is the ability of actinomycosis to mimic malignant or granulomatous conditions, often resulting in delayed diagnosis (11). In rare cases, *A. odontolyticus* has been identified in liver abscesses and other atypical intra-abdominal localizations (12).

In our patient, postoperative actinomycosis manifested as a subaponeurotic abscess following appendectomy and may have been associated with bacterial translocation in the setting of acute phlegmonous appendicitis; however, this mechanism remains hypothetical in the absence of direct intraoperative or microbiological confirmation. Although laparoscopic appendectomy is generally associated with a lower rate of surgical wound complications because of smaller incisions, reduced tissue trauma, and less exposure of the wound to external contamination, it does not completely eliminate the risk of postoperative infection. In this patient, the development of a postoperative abscess was considered more likely to be related to the underlying infectious-inflammatory process than to the choice of surgical approach itself.

The absence of positive blood cultures in this case should be interpreted in the clinical context. The patient exhibited no signs of systemic inflammatory response or sepsis; therefore, there were no clinical indications for blood culture sampling. Current clinical guidelines recommend blood cultures primarily in patients with suspected bacteremia or systemic infection, which was not observed in the present case.

Importantly, although the detection of *Actinomyces odontolyticus* in intra-abdominal or postoperative abscesses has been sporadically reported worldwide, this case represents the first documented isolation of this pathogen in the Republic of Kazakhstan. This epidemiological aspect is clinically relevant, as *A. odontolyticus* remains underdiagnosed in routine surgical practice, particularly in regions where advanced identification methods are not routinely available.

Conventional biochemical identification techniques have limited sensitivity for Actinomyces species due to their slow growth, fastidious nature, and phenotypic similarity to other anaerobic Gram-positive rods. As a result, such infections may remain unrecognized or be attributed to nonspecific polymicrobial flora. However, failure to identify Actinomyces spp. may be clinically significant, since delayed or inadequate etiological diagnosis can contribute to persistent infection, atypical wound healing, or recurrence (13). In contrast, MALDI-TOF mass spectrometry offers high diagnostic accuracy and has become increasingly adopted in clinical microbiology laboratories (14–16). Previous studies report species-level identification accuracy exceeding 95% for Actinomyces spp., highlighting the value of modern diagnostic approaches in detecting uncommon but clinically relevant pathogens (17).

In the present case, ceftriaxone was selected based on antimicrobial susceptibility testing and its availability in the clinical setting. The patient received intramuscular ceftriaxone during hospitalization, with a total treatment duration of 17 days. A prolonged course of therapy, which is often recommended in classical actinomycosis, was not considered necessary in this case, likely due to the localized nature of the infection and effective surgical source control. No oral step-down therapy was prescribed, as complete clinical and laboratory resolution was achieved. This case highlights that, in selected patients with localized postoperative actinomycosis and adequate drainage, shorter courses of targeted antimicrobial therapy may be sufficient.

Clinical improvement was observed following targeted antibacterial therapy with ceftriaxone, including resolution of fever and regression of local inflammatory signs. It should be noted that antimicrobial susceptibility testing for anaerobic bacteria is not routinely performed in many clinical microbiology laboratories. However, this is often less problematic for Actinomyces species, which are usually susceptible to beta-lactam antibiotics and, as Gram-positive organisms, are also generally sensitive to vancomycin (18). This case underscores the importance of combining adequate surgical source control with targeted antimicrobial therapy in the management of atypical postoperative infections caused by rare anaerobic pathogens.

## 4 Conclusion

This report describes the first documented case of postoperative abdominal actinomycosis caused by *Actinomyces odontolyticus* in Kazakhstan. Early microbiological diagnosis and antimicrobial susceptibility testing were pivotal for successful treatment. The case highlights the diagnostic value of MALDI-TOF mass spectrometry and the necessity of considering rare anaerobic pathogens in atypical postoperative infections. Even in the absence of systemic infection, microbiological examination of purulent material using modern identification techniques remains crucial for detecting uncommon pathogens and optimizing postoperative management.

## Data Availability

The original contributions presented in the study are included in the article/supplementary material, further inquiries can be directed to the corresponding author/s.
